# Optimal binning of continuous traits: Is the tradition tenable?

**DOI:** 10.3168/jdsc.2026-1064

**Published:** 2026-08-03

**Authors:** Xiao-Lin Wu, Guilhmere J.M. Rosa, Allicia Horn, Ezequiel Nicolazzi, John B. Cole

**Affiliations:** 1 Council on Dairy Cattle Breeding, Bowie, MD 20716; 2 Department of Animal and Dairy Sciences, University of Wisconsin, Madison, WI 53706; 3 Brown Swiss Association, Beloit, WI 53511; 4 Department of Animal Sciences, Donald Henry Barron Reproductive and Perinatal Biology Research Program, and the Genetics Institute, University of Florida, Gainesville, FL 32611; 5 Department of Animal Science, North Carolina State University, Raleigh, NC 27695; 6 Department of Animal Biosciences, University of Guelph, Guelph, ON, Canada N1G 2W1

## Abstract

Discretizing continuous traits is a standard practice in animal breeding. In dairy cattle, for example, conformation or linear-type traits derived from continuous measurements are commonly converted into categorical scores. Although binning procedures are often ad hoc, it remains unclear whether traditional binning conventions are statistically well justified or optimal. This study revisited and formalized the theoretical foundations of discretizing continuous variables and derived principled criteria for optimal bounded equal-interval binning, a widely used approach in the dairy industry. Within a standard normal framework, optimal bin widths (*δ*) and the number of bins (*k*) are derived either heuristically or through formally maximizing information retention. The latter is measured by the squared Pearson correlation (*ρ*^2^) between the original value and the conditional group means, or approximated by the correlation between the original value and the discretized scores. Closed-form expressions are obtained, allowing optimal bin widths and boundaries to be determined analytically. Numerical optimization further reveals systematic and predictable relationships among bin number, coverage span, and retained information. Under the Pearson-based optimization, approximately 5, 7, 9, and 20 bins retain 90%, 95%, 97%, and 99% of the original variance, respectively. Overall, 7 to 9 optimally spaced bins are sufficient to preserve ∼95% to 97% of the original information, whereas overly coarse binning introduces substantial and quantifiable information loss. Although traditional binning practices may retain adequate information at higher resolution, careful and principled calibration becomes essential when using coarse categorical scales. These findings provide a formal and quantitative benchmark for evaluating and optimizing traditional categorical scoring systems.

## Graphical Abstract

**Figure:**
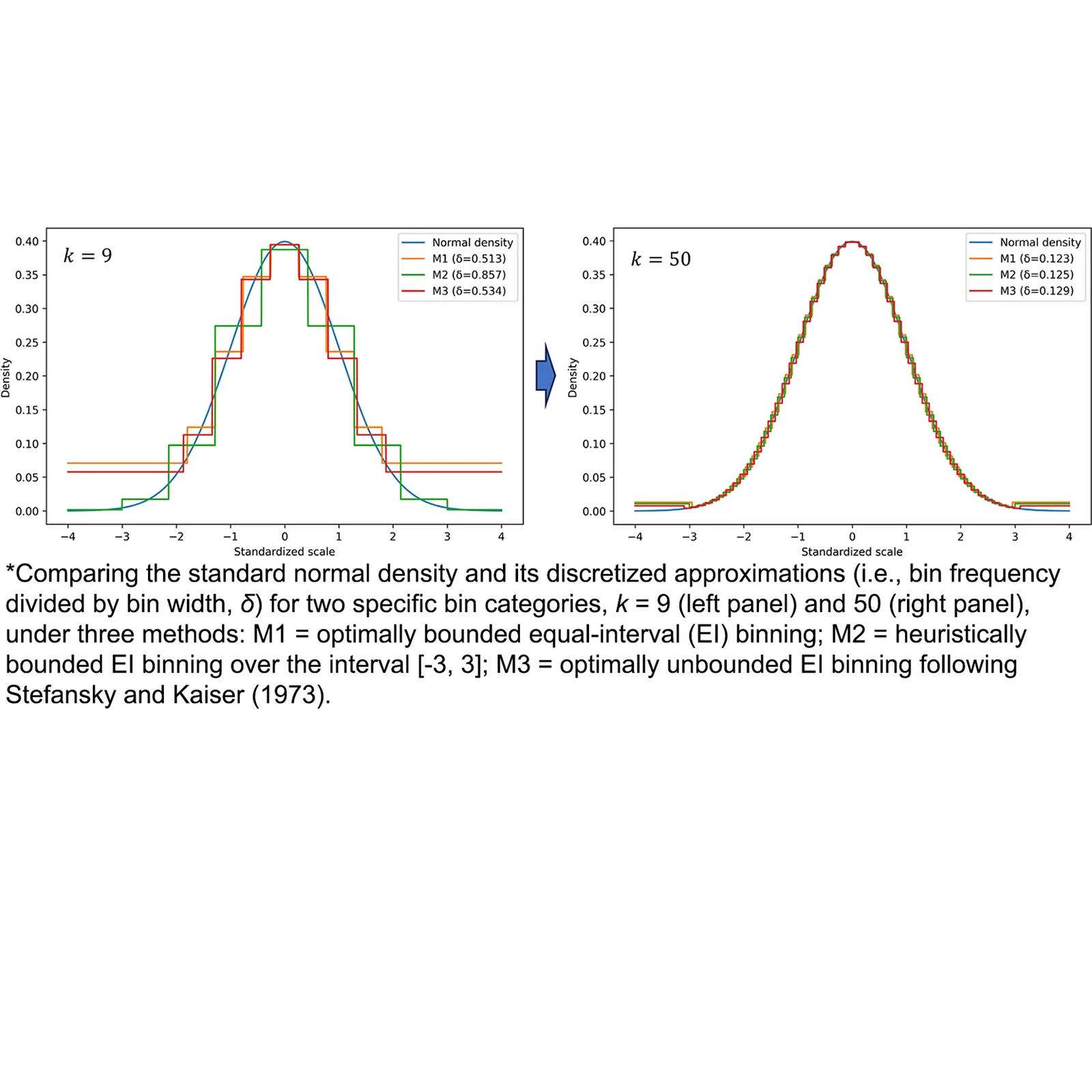

In dairy cattle breeding and management, conformation or linear-type traits are typically recorded on ordinal scales, most commonly 1 to 9. However, some systems extend to 50 or 80 categories (Wu et al., 2026). These traits exhibit moderate heritability and play an important role in selection programs (Veerkamp and Brotherstone, 1997; Manafiazar et al., 2016), serving as indicators of structural soundness, longevity, and lifetime productivity. Although many traits are visually assessed, others, such as stature, are first measured on a continuous scale and then discretized into categorical scores for use in genetic evaluation and management.

Generally, converting a continuous variable to a categorical variable entails partitioning its range into discrete intervals and assigning each observation to the interval it falls into (Cox, 1957). This process, commonly referred to as discretization or binning (also known as data chopping), results in an ordered set of categories, each representing an interval (or bin) corresponding to a classification level (Morrison and Toy, 1982).

Traditionally, equal-interval (**EI**) binning divides a variable into *k* intervals of equal width (denoted by *δ*) across the entire range of the distribution, from −∞ to +∞, and defines the thresholds as follows (Cox, 1957):

−∞ = *τ*_0_ < *z*_1_ < . . . < *z_k_*_−1_ < *z_k_* = ∞.

Mathematically, the interior thresholds are given as

[1] $$\text{\textbackslash{}[}z_{j}=\left(j\frac{k}{2}\right)\delta ,j=1,\ldots ,k-1,\text{\textbackslash{}]}$$

where *z_j_* denotes the *j*th threshold. In practice, this type of unbounded EI binning is rarely implemented due to the existence of outliers. Instead, bounded EI binning is often adopted, where bins are defined over a central range, with 2 outer categories capturing the extreme tails. Nevertheless, formal statistical principles for optimal bounded EI binning remain underdeveloped. In practice, bin widths are often chosen arbitrarily, driven more by historical practice and operational convenience than by statistical efficiency or biological interpretability. For instance, cattle stature is commonly discretized into one-inch intervals, which is practically convenient but not necessarily statistically optimal (Wu et al., 2026).

Motivated by these gaps, this study revisits and formalizes the theoretical foundations of discretizing continuous traits and develops principled criteria for optimal bounded EI binning. To maintain generality, we consider a standardized trait with mean zero and unit variance, noting that the results can be readily extended to the original scale through simple linear rescaling.

Let [−Λ, Λ] be a finite coverage range, where Λ > 0 denotes the truncation point on the standardized scale. This coverage range is then partitioned into *k* − 2 subintervals of equal width, with the bin width defined as $\delta =\frac{2\Lambda }{k-2},$ and the corresponding internal bin boundaries given by

[2] *z_j_* = −Λ + (*j* – 1)*δ*, for *j* = 1, . . ., *k* – 1.

The 2 extreme bins correspond to (−∞, −Λ] and [Λ, ∞), respectively.

This formulation provides the general framework for bounded EI binning. For a standard normal distribution, approximately 99.7% of observations lie within the interval [−3, 3], with a total width of 6. Hence, a commonly used heuristic approach is to set Λ = 3Λ, yielding the following bin width:

$$\delta =\frac{2\Lambda }{k-2}=\frac{6}{k-2}.$$

Choices of Λ also include Λ = 1.645, 1.96, and 2.576, corresponding to central coverage probabilities of 90%, 95%, and 99%, respectively, under the standard normal distribution. These choices are heuristic and do not necessarily ensure optimal information retention after discretization.

Statistically, optimal bin widths and the optimal number of bins should be defined by minimizing information loss induced by discretization. Under this unbounded setting, optimal bin widths have been derived by maximizing the squared Pearson correlation between continuous values and their discretized scores over the whole domain (i.e., from −∞ to +∞; Stefansky and Kaiser, 1973). However, the squared Pearson correlation based on discretized scores should be viewed as a proxy for information retention rather than an exact measure.

In what follows, we present a principled framework for optimal bounded EI binning. Let the representative values be the conditional group means. Then, the extent of information retained after discretization (M) can be quantified by the following variance ratio (Cox, 1957):

[3] $$M=\frac{Var\left[\xi \left(Y\right)\right]}{Var\left(Y\right)}=\frac{\sum_{j=1}^{k}p_{j}{\left(\xi_{j}-\bar{\xi }\right)}^{2}}{Var\left(Y\right)},$$

where Var(*Y*) denotes the variance of the original variable, $p_{j}=Pr\left(Y\in B_{j}\right)$ represents the probability mass in bin *B_j_*, *ξ*(*Y*) maps each observation to the conditional mean $\xi_{j}=E\left[Y|Y\in B_{j}\right],$ and $\bar{\xi }=\sum_{j=1}^{k}p_{j}\xi_{j}=E\left[Y\right]$ is the overall mean (corresponding to the mean of *Y*).

Therefore, information loss L, denoted as the proportion of total variance lost due to discretization, is given by

[4] $$L=\frac{E\left\{{\left[Y-\xi \left(Y\right)\right]}^{2}\right\}}{Var\left(Y\right)}=1-M.$$

Conceptually, L and M are complementary quantities. As the number of bins decreases (i.e., as bin width increases), between-group variation diminishes, leading to smaller M and larger L. In the extreme case where *k* = 1, all observations fall into a single bin, yielding M = 0 and L = 1, which corresponds to complete information loss. Conversely, as *k* increases and the bin widths become narrower, M → 1 and L → 0, indicating that the discretized variable increasingly approximates the original continuous scale with nearly complete information retention and negligible information loss.

Let Y* = *ξ*(*Y*) denote the discretized variable defined by conditional means, where *Y* ∼ *N*(0,1), obtained by replacing each observation with the conditional mean within its bin. For bin *B_j_* = (*z_j_*_−1_,*z_j_*] with probability $p_{j}=P\left(Y\in B_{j}\right)=\Phi \left(z_{j}\right)-\Phi \left(z_{j-1}\right),$ the conditional mean is

[5] $$\text{\textbackslash{}[}\xi_{j}=E\left[Y|Y\in B_{j}\right]=\frac{1}{p_{j}}\int_{z_{j-1}}^{z_{j}}y\phi \left(y\right)dy=\frac{\phi \left(z_{j-1}\right)-\phi \left(z_{j}\right)}{p_{j}},\text{\textbackslash{}]}$$

where Φ(·) and *ϕ*(·) denote the standard normal cumulative distribution function and probability density function, respectively. The final equality follows from the identity ∫yϕ(y)dy=−ϕ(y) and ϕ(±∞)=0.

Because Y* takes value *ξ_j_* with probability *p_j_*, and noting that E(Y)=E(Y∗)=0 under symmetry, the covariance and variance of Y* are

$$\begin{array}{c} Cov\left(Y,Y*\right)=E\left(Y,Y*\right)-E\left(Y\right)E\left(Y*\right)=\\ \sum_{j=1}^{k}p_{j}\xi_{j}\;E\left[Y|Y\in B_{j}\right]=\sum_{j=1}^{k}p_{j}\;\xi_{j}^{2}; \end{array}$$

$$\begin{array}{c} Var\left(Y^{*}\right)=E\left({\left(Y^{*}\right)}^{2}\right)-{\left[E\left(Y^{*}\right)\right]}^{2}=\\ \sum_{j=1}^{k}p_{j}\;\xi_{j}^{2}-{\left(\sum_{j=1}^{k}p_{j}\xi_{j}\right)}^{2}=\sum_{j=1}^{k}p_{j}\;\xi_{j}^{2}. \end{array}$$

Under symmetric binning around zero, $\sum_{j=1}^{k}p_{j}\xi_{j}=0,$ which simplifies these expressions. Therefore, the squared Pearson correlation between *Y* and its discretized counterpart Y* is

[6] $$\rho^{2}={\left(\frac{Cov\left(Y*,Y\right)}{\sqrt{Var\left(Y*\right)Var\left(Y\right)}}\right)}^{2}=\frac{Var\left(Y*\right)}{Var\left(Y\right)}=\sum_{j=1}^{k}p_{j}\;\xi_{j}^{2},$$

where Var(*Y*) = 1 and Cov(Y,Y∗)=Var(Y∗). Thus, when grouped values are defined as conditional means, the information retention measure M=Var[E(Y|B)]/[E(Y|B)]Var(Y)Var(Y) is exactly equal to the squared Pearson correlation between the original and discretized variables. This identity follows directly from the law of total variance and holds for any underlying distribution. Standardization and symmetry simplify the derivation but are not required for the result to hold.

Under bounded EI binning, the optimal bin width is determined by maximizing the information retention with respect to the span Λ > 0 for a fixed number of bins *k* > 2. Specifically, the bounded interval (−Λ,Λ) is partitioned into *k* − 2 equal-width interior bins, and the remaining 2 bins correspond to the lower and upper tails, (−∞,−Λ) and (Λ,∞), respectively. The width of each interior bin is therefore

$$\delta =\frac{2\Lambda }{k-2}.$$

Thus, the total number of bins is always *k*. When *k* is even, *k* − 2 is also even, so the origin (0) lies on the boundary between the 2 central interior bins. In contrast, when *k* is odd, *k* − 2 is odd, and the middle interior bin is centered at the origin. In the special case of binary binning (*k* = 2), there are no interior bins, and optimization over Λ is unnecessary because the procedure simply partitions the distribution at its median, thereby dividing the data into 2 equally probable halves.

Given a fixed number of bins *k* ≥ 3, the bin cutpoints are defined as

$$\text{\textbackslash{}[}\begin{array}{c} z_{0}=-\infty ,z_{j}=-\Lambda +\left(j-1\right)\frac{2\Lambda }{k-2}\\ forj=1,\ldots ,k-1,andz_{k}=+\infty . \end{array}\text{\textbackslash{}]}$$

To determine the optimal span Λ, derivatives with respect to Λ are required. For the standard normal density, using ϕ′(x)=−xϕ(x), the derivative of the cutpoints is

$$\text{\textbackslash{}[}a_{j}:=\frac{dz_{j}}{d\Lambda }=-1+\frac{2\left(j-1\right)}{k-2},j=1,\ldots ,k,\text{\textbackslash{}]}$$

$$with\frac{dz_{0}}{d\Lambda }=0,and\frac{dz_{k}}{d\Lambda }=0.$$

Define $<!--ERROR-14INMATHTYPECONVERSION.PLEASECORRECTMANUALLY-->\$\beta_{j}:=\phi \left(z_{j-1}\right)-\phi \left(z_{j}\right),\$$ such that $\xi_{j}=\frac{\beta_{j}}{p_{j}}.$ The derivatives of $p_{j}=\Phi \left(z_{j}\right)-\Phi \left(z_{j-1}\right)$ and $\beta_{j}=\phi \left(z_{j-1}\right)-\phi \left(z_{j}\right)$ are

$$\text{\textbackslash{}[}p_{j}^{\prime }=\phi \left(z_{j}\right)\;a_{j}-\phi \left(z_{j-1}\right)\;a_{j-1},\text{\textbackslash{}]}$$

$$\beta_{j}^{\prime }=\phi \prime \left(z_{j-1}\right)a_{j-1}-\phi \prime \left(z_{j}\right)a_{j}=-z_{j-1}\phi \left(z_{j-1}\right)\;a_{j-1}+z_{j}\phi \left(z_{j}\right)\;a_{j}.$$

Under symmetry around zero,

$$\text{\textbackslash{}[}z_{k-j}=-z_{j},a_{k-j}=-a_{j},p_{k+1-j}=p_{j},and\xi_{k+1-j}=-\xi_{j},\text{\textbackslash{}]}$$

which reduces the computational burden by approximately one-half.

For a fixed *k*, the Pearson-based information retention is $\rho^{2}=\sum_{j=1}^{k}p_{j}\xi_{j}^{2}=\sum_{j=1}^{k}\frac{\beta_{j}^{2}}{p_{j}}.$ Differentiating with respect to Λ gives

$$\frac{d}{d\Lambda }\left(\frac{\beta_{j}^{2}}{p_{j}}\right)=\frac{2\;\beta_{j}\;\beta_{j}^{\prime }\;p_{j}-\beta_{j}^{2}\;p_{j}^{\prime }}{p_{j}^{2}}.$$

Therefore, the optimal Λ (and therefore *δ*) satisfies the following first-order condition:

[7] $$\sum_{j=1}^{k}\frac{2\;\beta_{j}\;\beta_{j}^{\prime }\;p_{j}-\beta_{j}^{2}\;p_{j}^{\prime }}{p_{j}^{2}}=0.$$

Substituting the expressions for $\beta_{j}^{\prime }$ and $p_{j}^{\prime }$ in Equation 7, the optimal value of Λ can be obtained via a one-dimensional numerical search. The solution is typically unique, as *ρ*^2^ is empirically unimodal in Λ.

The procedure for obtaining the optimal solution is as follows: For a fixed *k*, systematically vary Λ and compute the corresponding cutpoints and bin probabilities; evaluate the objective function *ρ*^2^; and identify the value of Λ that maximizes the objective. The optimal equal bin width then follows as δ=2Λ/2Λ(k−2)(k−2).

First, we show how varying bin width influences information retention after discretization. Figure 1 illustrates the relationship between squared Pearson correlations *ρ*^2^ $\left(and\rho_{*}^{2}\right)$ and the equal-interval bin width *δ* for both bounded and unbounded EI binning with *k* = 9 categories. Here, *ρ*^2^ denotes the squared Pearson correlation between the original values and the conditional group means, which is the objective under bounded EI binning; $\rho_{*}^{2}$ denotes the squared Pearson correlation between the original values and the discretized scores, which is the objective under unbounded EI binning.

**Figure 1:**
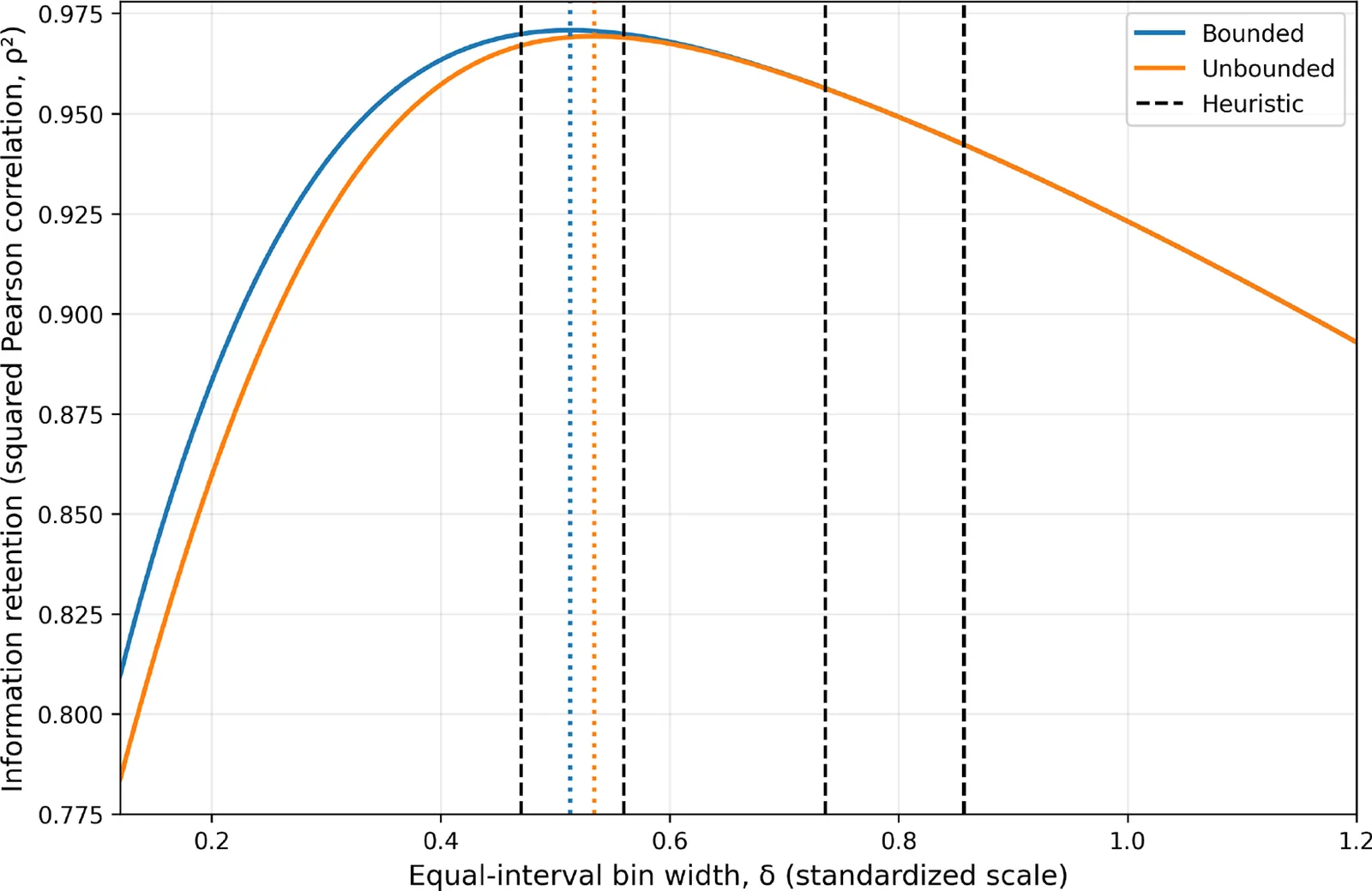
Squared Pearson correlation as a function of equal-interval bin width for bounded and unbounded binning approaches with *k* = 9, with heuristic bin width references based on central coverage probabilities of 90%, 95%, 99%, and 99.7% (black dotted lines). The blue and orange dotted lines represent the optimal bin widths for bounded and unbounded equal-interval binning, respectively.

Both curves exhibit a clear unimodal relationship: when *δ* is small, bins are too narrow within a limited span, leading to obvious truncation effects and reduced information retention; as *δ* increases, both *ρ*^2^ and $\rho_{*}^{2}$ increase as the bins better capture the distribution; beyond the optimum, further increases in *δ* produce overly coarse grouping, reducing resolution and causing both *ρ*^2^ and $\rho_{*}^{2}$ to decline. This pattern reflects the fundamental trade-off between within-bin homogeneity and between-bin discrimination.

The optimal bin widths differ slightly between bounded and unbounded EI binning. The bounded EI method reaches its maximum at *δ* ≈ 0.513 (Figure 1, blue dotted line), whereas the unbounded EI approach attains its optimum at *δ* ≈ 0.534 (Figure 1, orange dotted line). At these optima, the corresponding *ρ*^2^ values are very close, with the bounded EI curve achieving a slightly higher peak. This small but consistent difference reflects a subtle intrinsic difference between the 2 methods: bounded EI binning explicitly optimizes the finite span Λ and uses conditional means within bins that maximize information retention. In contrast, the unbounded EI approach distributes bins uniformly across the entire real line using fixed category scores, thereby maximizing the association between the original and discretized values. Thus, optimal bins under the unbounded scenario may not necessarily be optimal under the bounded scenario.

Statistically, the squared Pearson correlation between the observed phenotypes and conditional group means provides an exact measure of information retention. In contrast, the corresponding quantity between observed phenotypes and discretized scores is only an approximation measure. In bounded EI binning, each observation is replaced by the conditional mean within its bin (i.e.,Y∗=E[Y|B]). Under this construction, the objective function directly quantifies the proportion of variance preserved after discretization. In contrast, the unbounded EI approach of Stefansky and Kaiser (1973) aims to maximize the squared correlation between the original variable and assigned discrete scores over the entire (unbounded) support. Because these grouped values do not equal the corresponding conditional means, they are not optimal predictors of *Y* within bins, potentially resulting in a small but systematic loss of efficiency relative to the bounded EI approach. Moreover, the variance of assigned discrete scores increases with the number of bins, which is not on the same scale as the original continuous trait. Therefore, we recommend using the squared Pearson correlation between the observed phenotypic values and their corresponding conditional means within bins as the appropriate objective for evaluating and optimizing EI binning schemes.

The vertical dashed lines in Figure 1 represent heuristic choices of bin width based on central coverage probabilities of 90%, 95%, 99%, and 99.7% (black dotted lines ordered from left to right). These reference values provide a practical benchmark for commonly used rules of thumb. The first 2 heuristics (*δ* ≈ 0.470 and 0.560) lie close to the optimal region, with the 95% rule nearly coinciding with the peak of both curves. In contrast, the larger coverage-based heuristics (*δ* ≈ 0.736 and 0.857) fall well to the right of the optimum, where information retention has already begun to decline. This pattern indicates that heuristic choices based on overly wide coverage ranges (e.g., ±3*σ*), though commonly used in practice, may lead to suboptimal discretization when the number of bins is moderate.

Table 1 summarizes the behavior of optimal bounded EI binning across a wide range of category numbers and compares it with both a heuristic bounded approach based on the fixed range [−3, 3] and the unbounded EI method of Stefansky and Kaiser (1973). Several clear patterns emerge that are both theoretically informative and practically relevant.

**Table 1:** Optimal span range (Λ) and bin width (δ) for optimal bounded equal-interval (EI) binning across a varying number of groups (*k* = 3 − 100), compared with heuristic EI bounded over [−3, 3] and unbounded EI according to Stefansky and Kaiser (1973)^1^

*k*	Optimal bounded EI				Heuristic bounded EI [−3, 3]				Optimal unbounded EI		
	Λ	δ	*ρ^2^*	*ρ^2^_r_*	Λ	δ	*ρ^2^*	*ρ^2^_r_*	δ	ρ^2^_*_	*ρ^2^_r_*
3	0.612	1.224	0.810	0.864	3.000	6.000	0.029	0.008	1.224	0.810	0.864
4	0.982	0.982	0.883	0.915	3.000	3.000	0.653	0.752	0.996	0.881	0.913
5	1.235	0.823	0.920	0.940	3.000	2.000	0.746	0.674	0.843	0.918	0.937
6	1.424	0.712	0.941	0.955	3.000	1.500	0.842	0.837	0.733	0.939	0.952
7	1.572	0.629	0.955	0.965	3.000	1.200	0.893	0.881	0.651	0.953	0.962
8	1.694	0.565	0.964	0.971	3.000	1.000	0.923	0.915	0.586	0.963	0.969
9	1.796	0.513	0.971	0.976	3.000	0.857	0.942	0.936	0.534	0.969	0.974
10	1.883	0.471	0.976	0.980	3.000	0.750	0.955	0.951	0.491	0.974	0.978
20	2.392	0.266	0.993	0.994	3.000	0.333	0.991	0.990	0.279	0.992	0.993
30	2.649	0.189	0.996	0.997	3.000	0.214	0.996	0.996	0.199	0.996	0.996
40	2.819	0.148	0.998	0.998	3.000	0.158	0.998	0.998	0.156	0.998	0.998
50	2.945	0.123	0.999	0.999	3.000	0.125	0.999	0.999	0.129	0.998	0.998
60	3.044	0.105	0.999	0.999	3.000	0.103	0.999	0.999	0.110	0.999	0.999
70	3.170	0.093	0.999	0.999	3.000	0.088	0.999	0.999	0.096	0.999	0.999
80	3.230	0.083	0.999	0.999	3.000	0.077	0.999	0.999	0.086	0.999	0.999
90	3.290	0.075	0.999	0.999	3.000	0.068	0.999	1.000	0.077	0.999	0.999
100	3.340	0.068	1.000	1.000	3.000	0.061	0.999	1.000	0.071	1.000	1.000

1 *ρ^2^* = squared Pearson correlation between original values and group means as the phenotypes for individuals within each category; ρ^2^_*_ = squared Pearson correlation between original values and discretized scores as the phenotypes for individuals within each category; *ρ^2^_r_* = squared Spearman rank correlation between original and binned phenotypes.

First, under optimal bounded EI binning, the span Λ increases steadily with *k*, from approximately 0.612 at *k* = 3 to over 3.34 at *k* = 100, whereas the corresponding bin width *δ* decreases monotonically. This reflects an intuitive allocation principle: with more categories available, the binning scheme expands its coverage to include more of the tails while simultaneously refining resolution in the central region. As a result, both *ρ*^2^ and $\rho_{*}^{2}$ increase rapidly with *k*, approaching unity even for moderate values (e.g., *ρ*^2^ ≈ 0.993 at *k* = 20).

Second, the heuristic bounded EI approach over [−3, 3] performs poorly when *k* is small but improves as *k* increases. The deficiency is most pronounced at *k* = 3, where *ρ*^2^ is nearly negligible (0.029) because the central range is forced into a single wide bin. Even at *k* = 4 or 5, substantial information loss persists relative to the optimal solution. However, as *k* increases, the heuristic bin width *δ* = 6/(*k* − 2) decreases and approaches the optimal design. By *k* ≥ 30, the heuristic and optimal bounded EI results are virtually indistinguishable. This indicates that fixed-span heuristics can be adequate for fine discretization but unreliable for coarse grouping.

Third, the optimal unbounded EI binning yields results closely aligned with the optimal bounded EI solution, especially as *k* increases. For small *k*, minor differences are observed (for example, at *k* = 9, *ρ*^2^ = 0.971 under bounded EI versus $\rho_{*}^{2}=0.969$ under unbounded EI), but these gaps are small and diminish quickly with increasing *k*. This convergence occurs because as bin width decreases, the influence of boundary treatment becomes negligible, and both approaches effectively approximate the continuous distribution.

Determining the optimal number of groups is a central question in the binning of a continuous trait (Preston and Colman, 2000). In practice, using too few groups may oversimplify the underlying variation and result in substantial information loss, whereas using too many groups reduces interpretability and undermines the practical utility of discretization. In the present study, 4 commonly used targets were evaluated to reflect acceptable levels of information or ranking preservation: 0.90, 0.95, 0.97, and 0.99. These targets led to the required minimum bin number of 5, 7, 9, and 20, respectively, under both bounded and unbounded EI binning. These results indicate that moderate granularity, roughly 7 to 9 bins, is sufficient to preserve 95% to 97% of the original variance, whereas near-complete retention (≥99%) requires substantially finer partitioning.

Similar conclusions about optimal category number have emerged outside animal breeding. In psychometrics and market research, Green and Rao (1970) recommended 6 to 7 response categories, consistent with the well-known “magic number seven” in cognitive psychology (Miller, 1956; Symonds, 1924), which suggests diminishing gains in discrimination beyond 7 categories (Preston and Colman, 2000). Conversely, some researchers have advocated fewer categories, typically 4 to 7 (Lozano et al., 2008), for greater practical simplicity at a cost of increased information loss. These findings support using approximately 7 to 9 categories to balance interpretability and statistical fidelity.

To gain insight into how these methods work, graphical comparisons of the standard normal density and its discretized approximations under 3 binning strategies (optimal bounded, heuristic bounded, and optimal unbounded EI) for *k* = 9 and *k* = 50 are shown in the graphical abstract. For *k* = 9 (left panel), clear and meaningful differences are evident among the 3 methods. Optimal bounded EI (M1) provides the closest approximation to the normal density, particularly in the central region where most of the probability mass lies. Its stepwise representation aligns well with the peak and the curvature of the density, reflecting the use of conditional means and an optimized span. Optimal unbounded EI (M3) is also close but possibly slightly less accurate, especially near the peak and in the shoulders, because the assigned scores do not scale well with the original scale. In contrast, the commonly used heuristic bounded EI (M2) shows noticeable distortion: the bins are too wide, leading to a flattened peak and exaggerated tails. This results from fixing the span [−3, 3], allocating insufficient resolution to the central region when the number of bins is limited. Overall, at moderate granularity, the choice of binning method has a substantial impact on how well the discretized distribution approximates the original density.

For fine binning with *k* = 50 (graphical abstract, right panel), the differences among the 3 methods largely vanish. All 3 stepwise approximations closely describe the normal density across the entire range, with only minor discrepancies that are barely visible except at the 2 extreme tails. As the number of bins increases, the bin width becomes small, and each method effectively provides a fine-grained approximation to the continuous distribution. In this regime, the influence of the span choice (bounded vs. unbounded) and the representation (conditional means vs. fixed scores) becomes negligible. Even the heuristic method performs nearly as well as the optimal approaches, because increased resolution compensates for its suboptimal design.

Therefore, the impact of the binning strategy is most pronounced when the number of categories is small to moderate and diminishes as granularity increases. When *k* is limited (e.g., 9 categories), optimal design—particularly the use of conditional means and an appropriately chosen span—can substantially improve the fidelity. However, as *k* becomes large (e.g., 50 categories), all reasonable binning methods converge to similar performance, and simple heuristics become adequate in practice. This reinforces the study's key message: although traditional binning practices may be acceptable at high resolution, careful, principled design is essential when working with coarse categorical scales.

To conclude, we have derived principled criteria for optimal bounded equal-interval binning of continuous traits, providing a theoretical alternative to unbounded EI binning, and recommend quantifying information retention using the squared Pearson rank correlation between the original values and the conditional group means. Within a standardized normal framework, 7 to 9 optimally spaced bins are sufficient to preserve ∼95% to 97% of the original information, whereas increasing the number of categories beyond 20 yields only marginal additional benefit. These results provide a firm quantitative basis for widely used 1-to-9 scoring systems, indicating that such scales are statistically defensible when the bin width and coverage span are appropriately aligned with the underlying phenotypic variation.
